# Dragon (RGMb) induces oxaliplatin resistance in colon cancer cells

**DOI:** 10.18632/oncotarget.10338

**Published:** 2016-06-30

**Authors:** Ying Shi, Xiao-Xiao Huang, Guo-Bin Chen, Ying Wang, Qiang Zhi, Yuan-Sheng Liu, Xiao-Ling Wu, Li-Fen Wang, Bing Yang, Chuan-Xing Xiao, Hui-Qin Xing, Jian-Lin Ren, Yin Xia, Bayasi Guleng

**Affiliations:** ^1^ Department of Gastroenterology, Zhongshan Hospital, Xiamen University, Xiamen, China; ^2^ State Key Laboratory of Cellular Stress Biology, Xiamen University, Xiamen, China; ^3^ School of Biomedical Sciences, Faculty of Medicine, The Chinese University of Hong Kong, Hong Kong, China; ^4^ School of Biomedical Sciences Core Laboratory, The Chinese University of Hong Kong Shenzhen Research Institute, Shenzhen, China; ^5^ Department of Basic Medical Sciences, Institute of Neuroscience, Medical College of Xiamen University, Xiamen, China; ^6^ Xiamen Branch, Zhongshan Hospital, Fudan University, Xiamen, China

**Keywords:** Dragon, oxaliplatin resistance, colon cancer, JNK, p38 MAPK

## Abstract

Colorectal cancer (CRC) is one of the most commonly diagnosed cancers and a major cause of cancer mortality. Chemotherapy resistance remains a major challenge for treating advanced CRC. Therefore, the identification of targets that induce drug resistance is a priority for the development of novel agents to overcome resistance. Dragon (also known as RGMb) is a member of the repulsive guidance molecule (RGM) family. We previously showed that Dragon expression increases with CRC progression in human patients. In the present study, we found that Dragon inhibited apoptosis and increased viability of CMT93 and HCT116 cells in the presence of oxaliplatin. Dragon induced resistance of xenograft tumor to oxaliplatinin treatment in mice. Mechanistically, Dragon inhibited oxaliplatin-induced JNK and p38 MAPK activation, and caspase-3 and PARP cleavages. Our results indicate that Dragon may be a novel target that induces drug resistance in CRC.

## INTRODUCTION

Colorectal cancer (CRC) is the second leading cause of cancer-associated mortality in the world [1]. A number of molecular abnormalities have been associated with CRC including mutations in k-ras oncogenes; the inactivation of the tumor suppressor genes APC, p53 and DCC; mutations in the DNA mismatch repair regulators MLH1 and MSH2; and the dysregulation of DNA methylation, microsatellite stability, and non-coding RNAs [2–5]. Since 5-fluorouracil (5-FU) was first used as an adjuvant therapy in 1990, increasing number of molecules have been identified and used for targeted therapies. For example, epidermal growth factor receptor (EGFR) targeting has been used to treat advanced colon cancer [6, 7], and an anti-interleukin-6 (IL-6) receptor antibody has been used to suppress angiogenesis and inhibit CRC growth [8]. There is also an immunotherapy targeting cancer-associated CD43 glycoforms [9]. On the other hand, oxaliplatin-based chemotherapy is still the standard adjuvant therapy for advanced CRC and a first-line treatment option in cases of metastasis. Oxaliplatin causes cell death through its cytotoxic effects, by preventing DNA synthesis, replication and transcription [10]. The current systemic treatment for CRC includes oxaliplatin or irinotecan combined with 5-fluorouracil (5-FU) [11]. However, as a single agent, oxaliplatin has been found to be less effective against transforming cancerous cells, and the acquisition of drug resistance remains a major stumbling block [12]. The molecular mechanisms underlying oxaliplatin resistance following CRC chemotherapy are poorly understood, and thus deserve further investigation.

Dragon, also known as RGMb, is a member of the repulsive guidance molecule (RGM) family [13]. Dragon is a co-receptor for bone morphogenetic protein (BMP) signaling, which plays essential roles in many biological processes [14, 15]. Our previous study demonstrated that Dragon inhibits E-cadherin expression in renal tubular cells of injured kidneys [16]. It also negatively regulates IL-6 expression in macrophages via the p38 and Erk1/2 MAPK pathways but not the Smad1/5/8 pathway [17]. Our recent study showed that Dragon expression increases with CRC progression [18]. In the present study, we examined the role of Dragon in the resistance of colon cancer cells to oxaliplatin treatment. We found that Dragon induced the resistance of CRC cells to oxaliplatin both *in vitro* and *in vivo*.

## RESULTS

### Dragon overexpression increases the resistance of colon cancer cells to oxaliplatin

**Figure 1:**
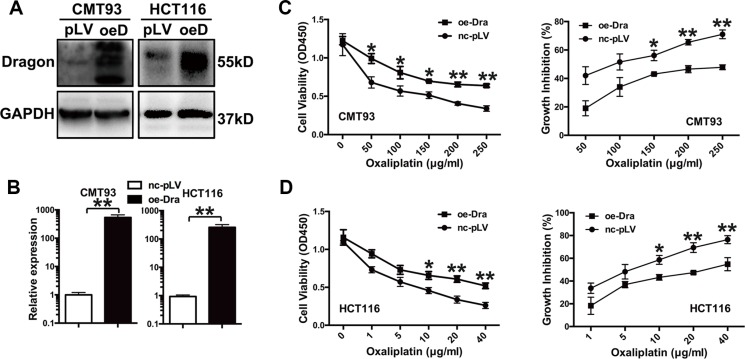
Dragon-overexpression induces the resistance of colon cancer cells to oxaliplatin *in vitro* (**A** and **B**) Expression of Dragon in Dragon-overexpressing CMT93 and HCT116 cells. Control (pLV or nc-pLV) and Dragon-overexpressing (oeD or oe-Dra) CMT93 and HCT116 cells were lysed to examine Dragon protein expression by Western blot analysis (A) and Dragon mRNA expression by qRT-PCR (B). GAPDH was used as a control. (**C**) Effect of Dragon overexpression on the viability and growth rate of CMT93 cells cultured in the presence of oxaliplatin. CCK8 assays were used to determine the viability (left panel) and growth inhibition rate (right panel) of control and Dragon-overexpressing CMT93 cells in the presence of increasing doses of oxaliplatin. (**D**) Effect of Dragon overexpression on the viability (left panel) and growth rate (right panel) of HCT116 cells in the presence of oxaliplatin. *P < 0.05 and **P < 0.01.

To examine the effects of Dragon on the cellular response to oxaliplatin, stable Dragon-overexpressing CMT93 and HCT116 cells were generated (Figure 1A and 1B). We then treated glucose-deprived (1.0 g/l) Dragon-overexpressing CMT93 cells with increasing concentrations of oxaliplatin (0 to 250 μg/ml) for 24 h. Cells were cultured in a glucose deprivation condition in order to simulate the tumor growth environment in human body. As determined by the CCK-8 assay, oxaliplatin reduced cell viability in both pLV-control and Dragon-overexpressing cells in a dose-dependent manner. However, Dragon-overexpressing cells showed higher viability than control cells (Figure 1C, left panel). The growth inhibition rates in Dragon-overexpressing cells were 45.3% at 50 μg/ml, 66.0% at 100 μg/ml, 76.8% at 150 μg/ml, 71.1% at 200 μg/ml and 67.4% at 250 μg/ml of oxaliplatin, of those in the respective pLV-controls (Figure 1C, right panel).

We also used human HCT116 colon cancer cells to confirm the results obtained with the murine CMT93 cells. We treated glucose-deprived control and Dragon-overexpressing HCT116 cells with increasing amounts of oxaliplatin (0 to 40 μg/ml) for 24 h. Dragon-overexpressing HCT116 cells showed a higher viability than control cells (Figure 1D, left panel). The growth inhibition rates in Dragon-overexpressing HCT116 cells were 54.4% at 1 μg/ml, 76.5% at 5 μg/ml, 73.8% at 10 μg/ml, 68.3% at 20 μg/ml and 71.8% at 40 μg/ml of oxaliplatin, of those in the corresponding pLV-controls (Figure 1D, right panel).

To further elucidate the effects of Dragon on growth inhibition by oxaliplatin in colon cancer cells, we performed a colony formation assay. As shown in Supplementary Figure S1A, Dragon overexpression increased cancer cell colony formation compared to pLV-control cells cultured in the presence of oxaliplatin and high glucose (4.5 g/l) for 7 days. The oxaliplatin-resistance rates in the control CMT93 cells were 25.5% and 43.5% in the presence of 2.5 and 5 μg/ml of oxaliplatin, respectively. However, the corresponding resistance rates fell to 14.9% and 36.1% in Dragon-overexpressing CMT93 cells (Supplementary Figure S1B). All of these results indicate that Dragon overexpression induces resistance of colon cancer cells to oxaliplatin.

### Dragon silencing decreases the resistance of colon cancer cells to oxaliplatin

**Figure 2:**
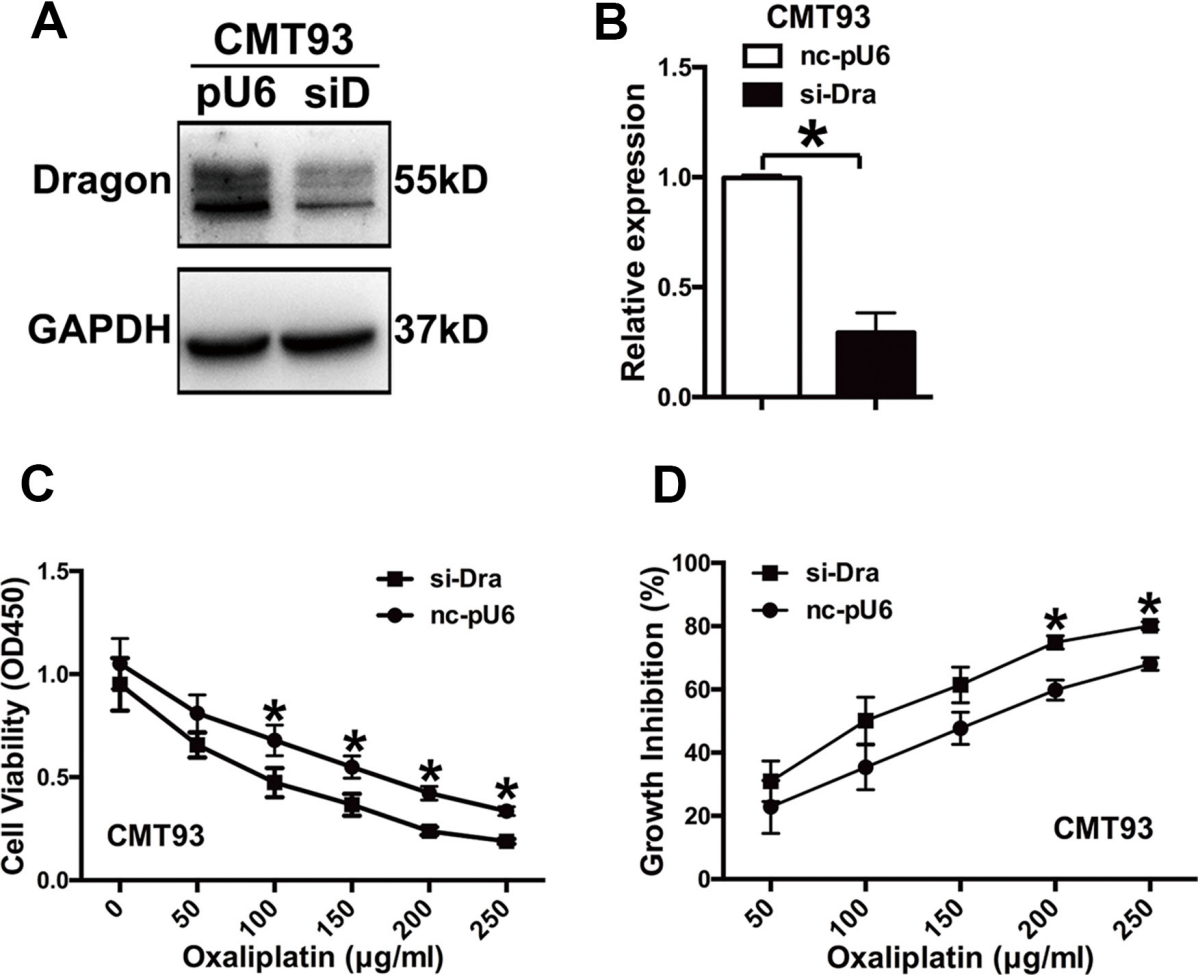
Inhibition of Dragon expression sensitizes CMT93 cells to oxaliplatin (**A** and **B**) Expression of Dragon in Dragon knockdown CMT93 cells. Control (pU6 or nc-pU6) and Dragon-knockdown (siD or si-Dra) CMT93 cells were lysed to examine Dragon protein expression by Western blot analysis (A) and Dragon mRNA expression by qRT-PCR (B). GAPDH was used as a control. (**C** and **D**) Effect of Dragon knockdown on the viability and growth rate of CMT93 cells in the presence of oxaliplatin. CCK8 assays were used to determine the viability (C) and growth inhibition rate (D) in control and Dragon knockdown CMT93 cells in the presence of increasing doses of oxaliplatin. *P < 0.05.

To corroborate the results obtained from Dragon overexpression, we generated stable Dragon-knockdown CMT93 cells (Figure 2A and 2B). We treated glucose-deprived Dragon-knockdown CMT93 cells with various concentrations of oxaliplatin (0 to 250 μg/ml) for 24 h. The Dragon-knockdown cells showed decreased viability as compared with control cells cultured in the presence of oxaliplatin (Figure 2C). The growth inhibition rates of the Dragon-knockdown CMT93 cell were 1.35 times at 50 μg/ml, 1.42 times at 100 μg/ml, 1.29 times at 150 μg/ml, 1.25 times at 200 μg/ml and 1.18 times at 250 μg/ml of oxaliplatin higher than those of the corresponding pU6-controls (Figure 2D).

Dragon knockdown also decreased cancer cell colony formation compared to pU6-control cells cultured in the presence of oxaliplatin for 7 days (Supplementary Figure S1C). The oxaliplatin-induced growth inhibition rates were 40.7% and 74.4% in pLV-control CMT93 cells in the presence of 2.5 and 5 μg/ml of oxaliplatin respectively. These rates increased to 59.9% and 93.1% in the corresponding Dragon knockdown cells (Supplementary Figure S1D). These results indicate that Dragon knockdown reduces oxaliplatin resistance in colon cancer cells.

### Dragon overexpression inhibits oxaliplatin-induced apoptosis in colon cancer cells

**Figure 3:**
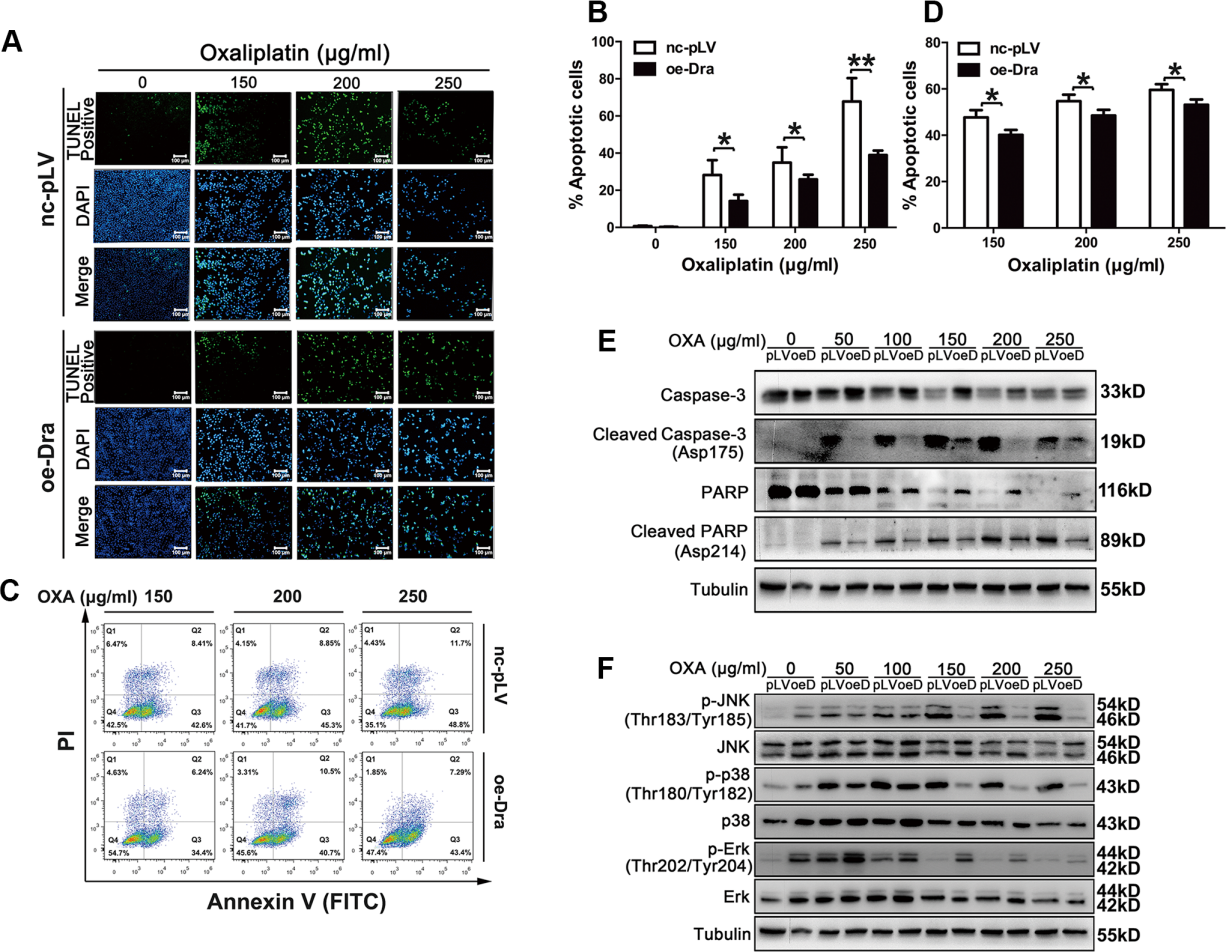
Dragon overexpression inhibits oxaliplatin-induced apoptosis in CMT93 cells (**A** and **B**) Glucose-deprived control (nc-pLV) and Dragon-overexpressing (oe-Dra) CMT93 cells were treated with increasing doses of oxaliplatin for 24 h before the TUNEL assay was performed. TUNEL-positive cells are shown in green, and the nuclei are shown in blue (A). The percentages of TUNEL-positive cells over total cell numbers are presented (B). (**C** and **D**) Control and Dragon-overexpressing CMT93 cells cultured in low-glucose medium were treated with increasing doses of oxaliplatin for 24 h before the cells were stained with AnnexinV-FITC/PI. Cell apoptosis was analyzed by flow cytometry (C). The percentages of apoptotic cells is shown (D). The data are presented as the mean ± SD of three independent experiments. *P < 0.05 and **P < 0.01. (**E**) Effect of Dragon overexpression on cleaved caspase-3 and cleaved PARP levels in CMT93 cells. Control (pLV) and Dragon overexpressing (oeD) CMT93 cells cultured in low glucose medium were treated with increasing doses of oxaliplatin for 24 h before the cells were harvested for Western blotting to detect cleaved caspase-3, full length caspase-3, cleaved PARP and full length PARP. (**F**) Effect of Dragon-overexpression on JNK, p38 and Erk phosphorylation in the presence of oxaliplatin under a glucose deprivation condition. Control (pLV) and Dragon-overexpressing (oeDra) CMT93 cells cultured in low glucose medium were treated with increasing doses of oxaliplatin for 24 h before the cells were collected for Western blotting to detect p-JNK/JNK, p-p38/p38 and p-Erk/Erk.

To determine the cellular mechanisms underlying the Dragon-induced resistance to oxaliplatin, we examined cell apoptosis in Dragon-overexpressing cells by the TUNEL assay and flow cytometry (Figures 3A–3D). Glucose-deprived control and Dragon-overexpressing CMT93 cells were treated with different doses of oxaliplatin for 24 h before the apoptosis assays were performed. As shown by the TUNEL assay (Figures 3A and 3B), oxaliplatin dramatically increased the number of TUNEL-positive control CMT93 cells, but these increases were significantly attenuated in Dragon-overexpressing cells. Flow cytometry also showed less apoptosis in Dragon-overexpressing CMT93 cells than in control cells (Figures 3C and 3D). Control and Dragon-overexpressing HCT116 cells were also analyzed for cell apoptosis. Flow cytometry revealed less apoptosis in Dragon-overexpressing HCT116 cells than in control cells (Supplementary Figure S2).

We then analyzed cleaved caspase-3 and cleaved-PARP, markers for apoptosis in glucose-deprived control and Dragon-overexpressing CMPT93 cells in the presence of oxaliplatin for 24 h. Cleaved caspase-3 and cleaved PARP were increased following oxaliplatin treatment in control CMT93 cells, and their levels were much reduced in Dragon overexpressing CMT93 cells (Figure 3E).

### Dragon silencing promotes oxaliplatin-induced apoptosis in CMT93 cells

**Figure 4:**
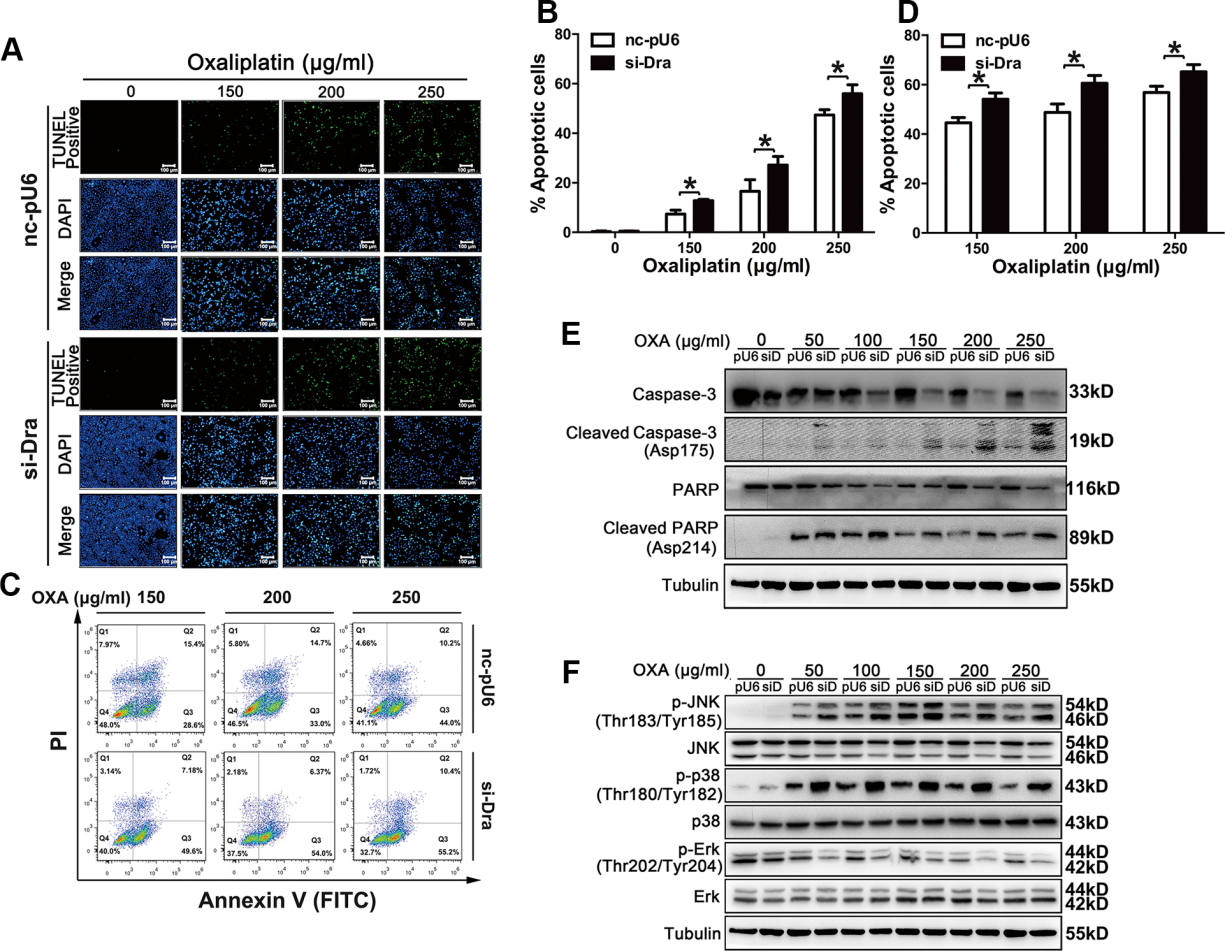
Dragon knockdown increases oxaliplatin-induced apoptosis in CMT93 cells (**A** and **B**) Control (nc-pU6) and Dragon knockdown (si-Dra) CMT93 cells cultured in low glucose medium were treated with increasing doses of oxaliplatin for 24 h before the TUNEL assay was performed. TUNEL-positive cells are shown in green, and the nuclei are shown in blue (A). The percentages of TUNEL-positive cells is presented (B). (**C** and **D**) Control and Dragon-knockdown CMT93 cells cultured in low glucose medium were treated with increasing doses of oxaliplatin for 24 h before the cells were stained with AnnexinV-FITC/PI. Cell apoptosis was analyzed by flow cytometry (C). The percentages of apoptotic cells is shown (D). The data are presented as the mean ± SD of three independent experiments. *P < 0.05. (**E**) Effect of Dragon-knockdown on cleaved caspase-3 and cleaved PARP levels in CMT93 cells. Control (pU6) and Dragon-knockdown (siD) CMT93 cells cultured in low glucose medium were treated with increasing doses of oxaliplatin for 24 h before the cells were harvested for Western blotting to detect cleaved caspase-3, full-length caspase-3, cleaved PARP and full-length PARP. Tubulin was used as a loading control. (**F**) Effect of Dragon knockdown on JNK, p38 and Erk phosphorylation in the presence of oxaliplatin. Control (pU6) and Dragon-knockdown (siD) CMT93 cells cultured in low glucose medium were treated with increasing doses of oxaliplatin for 24 h before the cells were collected for Western blotting to detect p-JNK/JNK, p-p38/p38 and p-Erk/Erk. Tubulin was used as a loading control.

The number of TUNEL-positive cells was significantly increased in Dragon-knockdown cells compared to that in control CMT93 cells (Figures 4A and 4B). Flow cytometry also showed more apoptosis in Dragon-knockdown CMT93 cells than in control cells (Figures 4C and 4D). Oxaliplatin-induced cleaved caspase-3 and cleaved PARP were increased in Dragon knockdown cells (Figure 4E). All of these results suggest that Dragon inhibits oxaliplatin-induced apoptosis.

### Dragon inhibits oxaliplatin-induced JNK and p38 MAPK activation in CMT93 cells

To investigate the molecular pathways involved in the Dragon-induced resistance to oxaliplatin in colon cancer cells, we analyzed the MAPK pathways in Dragon-overexpressing or knockdown cells. The phosphorylation levels of JNK and p38, but not Erk, in control CMT93 cells were increased by 24 hrs oxaliplatin treatment. Dragon overexpression inhibited the oxaliplatin-stimulated phosphorylation of JNK and p38MAPK (Figure 3F). Conversely, Dragon knockdown increased the levels of oxaliplatin-induced JNK and p38 MAPK phosphorylation (Figure 4F). Therefore, the inhibition of apoptosis by Dragon is associated with decreased JNK and p38 MAPK activities.

### Dragon induces oxaliplatin resistance *in vivo*

**Figure 5:**
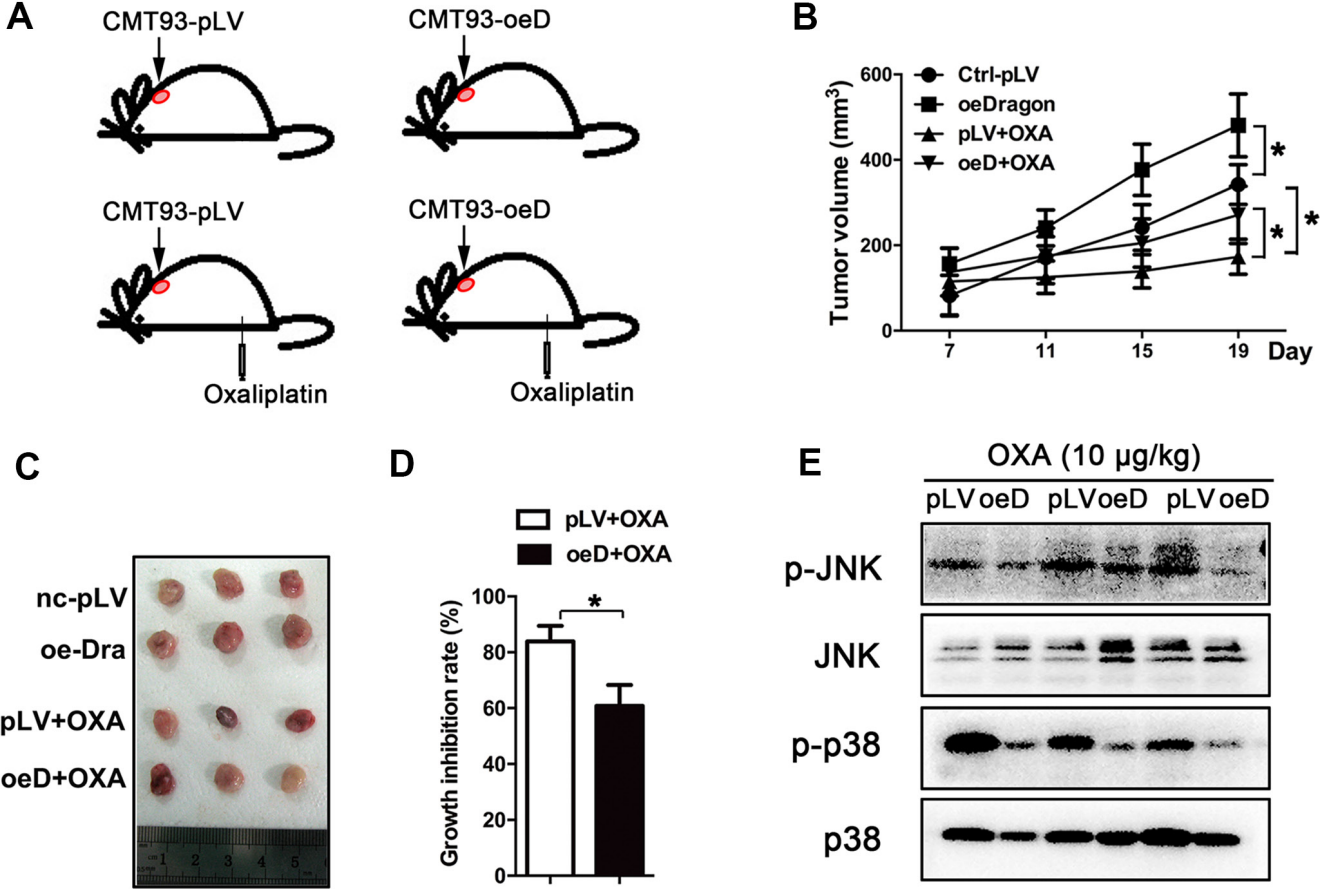
Dragon-overexpression induces the resistance of xenograft tumors to oxaliplatin *in vivo* (**A**–**D**) Effect of Dragon-overexpression on xenograft tumor growth. C57/BL6 mice were subcutaneously injected with control (pLV) or Dragon-overexpressing (oeD) CMT93 cells. Seven days after cell injection, oxaliplatin (10 mg/kg) was intraperitoneally injected into the mice once every 3-4 days (A). Xenograft tumor volumes were measured once every 4 days using a caliper (B). Nineten days after cell injection, the xenograft tumors were dissected and presented (C). The tumor growth inhibition rates were calculated (D). *P < 0.05. (**E**) Effect of Dragon overexpression on JNK and p38 phosphorylation in mouse xenograft tumors treated with oxaliplatin. Control (pLV) and Dragon-overexpressing (oeD) xenograft tumors derived from CMT93 cells treated with oxaliplatin were collected for Western blotting to detect p-JNK/JNK and p-p38/p38

To investigate the role of Dragon in oxaliplatin sensitivity *in vivo*, synchronized Dragon-overexpressing or control CMT93 cells were injected subcutaneously into C57/BL6 mice. Seven days later when the xenograft tumors were well-formed, the tumor-bearing mice were intraperitoneally injected with oxaliplatin (10 mg/kg) once every 3–4 days for 11 days (Figure 5A). The tumors were dissected and weighed every 4 days (Figure 5B). Tumor growth was slightly greater in the Dragon-overexpressing cells than in control CMT93 cells in the absence of oxaliplatin. As expected, tumor growth was inhibited by oxaliplatin in control CMT93 cells. Tumors derived from Dragon overexpressing CMT93 cells were significantly larger than those from control cells treated with oxaliplatin (Figures 5C). The oxaliplatin-mediated growth inhibition rate of the control tumors was 83.83 ± 5.61%, and this number was significantly reduced to 60.82 ± 7.50% in Dragon-overexpressing tumors (Figure 5D).

We analyzed the BMP signaling pathways in the xenograft tumors collected at day 19 after cell injection. Coupled with the changes in tumor sizes, oxaliplatin-induced JNK and p38 MAPK phosphorylation in the tumor tissues was lower in tumors from Dragon-overexpressing CMT 93 cells than in tumors from control CMT93 cells (Figure 5E). Smad1/5/8 phosphorylation was higher in Dragon overexpressing tumors than in control tumors following treatment with oxaliplatin (Supplementary Figure S3). These results suggest that Dragon inactivates the JNK and p38 MAPK pathways and blunts the sensitivity of tumors to oxaliplatin *in vivo*.

## DISCUSSION

In our previous study, we demonstrated that Dragon expression increased in colon cancers, especially in those at advanced stages, compared with control tissues, and that Dragon promoted colon cancer cell proliferation *in vitro* and CRC progression *in vivo* [18]. In the present study, we discovered another important role for Dragon in the inhibitions of oxaliplatin-induced CRC cell apoptosis and the subsequent resistance of CRC cells to oxaliplatin treatment.

In our assays for cell viability and apoptosis, we cultured cells in medium with a very low glucose concentration to minimize cell proliferation. Therefore, Dragon's effects on cell proliferation, which were found to be significant in cells cultured in high glucose concentrations [16], are presumably minor in our low glucose condition. Importantly, the reduction in cell apoptosis following Dragon overexpression or the increase in cell apoptosis upon Dragon knockdown was similar to the increase or decrease in cell viability that was observed following Dragon overexpression or knockdown respectively. These results suggest that the increase in cell viability in the presence of oxaliplatin caused by Dragon is largely attributed to the inhibitory effects of Dragon on cell apoptosis.

One of the chemotherapeutic strategies for cancer treatment is to increase cell apoptosis. The sensitivity of cancer cells to chemotherapy-induced apoptosis is regulated by a variety of factors including gene mutations and altered gene expression. For example, it has been shown that Ras mutations promoted apoptosis in response to 5-FU treatment [19]. Fibroblast growth factor receptor 4 (FGFR4) was found to be highly expressed in colon cancers and to induce drug resistance [20]. Here we identified Dragon as another drug resistance-inducing gene. Whether Dragon is targetable in treating chemo-resistance in CRC remains unknown, but is worth further investigation.

Our previous study demonstrated that Dragon inhibited the expression of IL-6 in macrophages [17]. Interestingly, our cytokine antibody array for the multiplex analysis of 48 cytokines demonstrated that Jam-a was downregulated in Dragon overexpressing xenograft tumors as compared with control tumors treated with oxaliplatin for 19 days (Supplementary Figure S4). Jam-a has been found to be dysregulated in some cancers. This dysregulation is associated with the outcome of certain cancers and might be a prognostic indicator. Low Jam-a expression was correlated with poor prognosis in gastric cancer, pancreatic cancer and breast cancer and was also positively associated with the sensitivity of multiple myeloma cells to chemotherapeutic drugs [21–23]. All of these previous observations suggest an inhibitory role for Jam-a in cancer growth. In the present study, we found that xenograft tumors derived from Dragon-overexpressing colon cancer cells grew faster than those from control cells in the presence of oxaliplatin and that Jam-a expression was downregulated in xenograft tumors derived from Dragon-overexpressing cells. These results are consistent with the role of Jam-a in inhibiting cancer growth. Further studies are needed to determine whether Dragon directly regulates Jam-a and whether Jam-a indeed plays a role in Dragon-induced resistance to oxaliplatin.

It is well documented that the JNK, Erk and p38 MAPK pathways regulate cell apoptosis and survival [24]. Under physiological conditions, activated Erk phosphorylates a number of kinases and transcription factors that execute programs related to cell cycle progression, differentiation, protein translation and evasion of cell death [25, 26]. The JNK and p38 MAPK pathways control cellular senescence and oncogenic transformation and modulate the cellular programs for survival and differentiation during the development of various cancers [27–29]. p38 MAPK inhibitors are currently in clinical trials for chronic inflammatory diseases [30]. p38β has been proposed to have anti-apoptotic effects in various cell lines and might counteract the pro-apoptotic effect of p38α [31]. p38α is required for colorectal cancer cell homeostasis as inhibition of its kinase function by pharmacological blockade or genetic inactivation causes cell cycle arrest, autophagy and cell death in a cell type-specific manner [32]. JNK is required for tumor cell survival. Because the absence of JNK stimulated apoptosis in Ras-induced JNK-null tumors, the use of JNK inhibitors as anti-cancer therapeutics was proposed. In some other settings, however, JNK activates apoptosis by interacting with the Bcl2 family of proteins [33]. Therefore, it appears that JNK may either promote or suppress tumor development depending on the settings. We previously found that in CRC, Dragon increased tumor progression by activating the Erk1/2 and Smad 1/5/8 pathways [18]. In the present study, oxaliplatin activated the p38 MAPK and JNK pathways. In this setting, Dragon overexpression blocked p38 MAPK and JNK activation while Dragon inhibition enhanced their activities. Thus, the JNK and p38 pathways may mediate the activity of Dragon in inducing oxaliplatin-resistance in CRC.

Taken together, these data revealed that Dragon inhibited oxaliplatin-induced JNK and p38 MAPK phosphorylation and reduced oxaliplatin-induced apoptosis in CRC cells. Dragon overexpression led to oxaliplatin resistance in xenograft mouse tumors.

## MATERIALS AND METHODS

### Ethics statement

This study was approved by the Ethics Committee of Zhongshan Hospital, Xiamen University (Xiamen, Fujian Province, China) (No. 20081009). All of the procedures involving experimental animals were performed in accordance with protocols that were approved by the Committee for Animal Research of Xiamen University and complied with the Guide for the Care and Use of Laboratory Animals (NIH publication No. 86-23, revised in 1985).

### Cell culture

CMT93 cells (purchased from ATCC, Manassas, VA, USA) were cultured in Dulbecco's modified Eagle's medium (DMEM) supplemented with 10% fetal bovine serum (Life Technologies, Grand Island, NY, USA) and 1% penicillin G/streptomycin. HCT116 cells (purchased from ATCC, Manassas, VA, USA) were cultured in McCoy's 5A (modified) medium supplemented with 10% fetal bovine serum (Life Technologies, Grand Island, NY, USA) and 1% penicillin G/streptomycin. Cells were maintained in a CO_2_ AutoZero incubator (Thermo, Massachusetts, USA) at 37°C with 5% CO_2_.

### Measurement of mRNA expression

Total RNA was extracted from cells using TRIzol (Invitrogen, Carlsbad, CA, USA) following the manufacturer's instructions. First-strand cDNA was synthesized using the RevertAid first-strand cDNA synthesis kit (Thermo Scientific, Fermentas, Lithuania). Mouse RGMb transcripts were amplified using primers described previously [34, 35].

### Western blotting

Proteins from total cell lysates or tumor tissues were separated by standard SDS-PAGE and then transferred to polyvinylidene fluoride (PVDF) membranes. The membranes were washed, blocked and incubated with primary antibodies against p44/42 MAPK (Erk1/2, #4695), phospho-p44/42 MAPK (Erk1/2, Thr202/Tyr204, #4370), p38 MAPK (#9212), phospho-p38 MAPK (Thr180/Tyr182, #9211), SAPK/JNK (#9258), phospho-SAPK/JNK (Thr183/Tyr185, #9211), caspase-3 (#9665), cleaved PARP (Asp214, #9544) and PARP (#9542) all purchased from Cell Signaling Technology (Boston, MA, USA). The primary antibody against Dragon (AF3597, AF3630), and the anti-sheep (HAF016) or anti-goat (HAF109) secondary antibodies were purchased from R&D Systems (Minneapolis, MN, USA).

### Cell viability and growth inhibition

The cell Counting Kit-8 (CCK-8, DoJinDo, Tokyo, Japan) was used to assess cell viability. Dragon-overexpressing or knockdown cells and the respective controls were seeded at a density of 8 × 10^3^ cells per well in 96-well plates. 24 h later, the cells were treated with or without oxaliplatin for 24 h. Absorbance at 450 nm was measured to determine cell viability. Growth inhibition = [cell viability without oxaliplatin - cell viability with oxaliplatin] / cell viability without oxaliplatin × 100%.

### Colony formation assays

Cells were plated in 6-well plates (Thermo, Massachusetts, USA) at a density of 1 × 10^3^ cells/well and treated with different concentrations of oxaliplatin for 7 days. The cultures were given fresh medium every 3 days. Seven days later, the colonies were fixed with 4% paraformaldehyde and then stained with 1% crystal violet. The colonies containing over 50 cells were counted. Growth inhibition = [colony number without oxaliplatin - colony number with oxaliplatin] / colony number without oxaliplatin × 100%.

### TUNEL assay

TdT-mediated dUTP nick-end labeling (TUNEL) assays were performed with the one-step TUNEL kit (Beyotime Institute of Biotechnology, China) following the manufacturer's instructions. Cells were cultured on poly-(L-lysine)-coated coverslips in 12-well culture clusters (Thermo, Massachusetts, USA) in low glucose DMEM (Gibco, Life Technologies, NY, USA) and treated with different concentrations of oxaliplatin. 24 h later, cells were fixed with 4% paraformaldehyde. The cells were then permeabilized with 0.1% Triton X-100 before photophobic incubation in 50 μl TUNEL reaction mixture for 1 h at 37°C. Finally, isometric DAPI was added, and the cells were incubated for 2 min at room temperature.

### Flow cytometry

Apoptosis was evaluated using the Annexin V-FITC Apoptosis Detection Kit I (BD, New Jersey, USA), according to the manufacturer's instructions. Cells were cultured in low glucose DMEM and treated with oxaliplatin for 24 h. The cells were collected and suspended in 500 μl of binding buffer. Five microliters of Annexin V-fluorescein isothiocyanate and 2.5 μl of PI were added, and the cell suspension was incubated at room temperature for 10 min. The samples were subjected to flow cytometry (Gallios, Beckman, America). The data were analyzed using Summit software (FlowJo, USA).

### Treatment of mouse xenograft tumors with oxaliplatin

Control or Dragon-overexpressing CMT93 cells (5 × 10^6^; CMT93-Control and CMT93-oeDragon, respectively) were subcutaneously injected into C57BL/6 mice. Seven days later, the mice were intraperitoneally injected with or without oxaliplatin at a dose of 10 mg/kg body weight once every 3–4 days (*n* = 6). Tumor sizes were measured every 4 days. Tumor growth inhibition = [volume of tumor without oxaliplatin treatment - volume of tumor with oxaliplatin treatment] / volume of tumor without oxaliplatin treatment × 100%.

### Cytokine antibody array

Proteins were extracted from Dragon-overexpressing or control xenograft tumors. Mouse cytokine array membranes (AAM-CYT-5-4, RayBiotech, Norcross, USA) were blocked for 30 min and then incubated with the protein samples at 4°C overnight. The samples were then removed, and the membranes were washed before they were incubated with biotin-conjugated antibodies at room temperature for 1 hr. After several washes, the membranes were incubated with HRP-conjugated streptavidin (1:1000 dilution) at room temperature for 2 hrs. The membranes were then washed thoroughly and exposed to detection buffer in the dark before being exposed to X-ray film. The intensities of signals were quantified by densitometry. The relative expression levels of the cytokines were determined. The positive controls were used to normalize the results from different membranes. The fold changes in protein expression were calculated.

### Statistical analysis

Statistical analysis was performed using SPSS 17.0 software (SPSS Inc., Chicago, IL, USA). Student's *t*-test was used when two groups were compared. All of the values are expressed as the mean ± SD of at least three independent experiments performed in triplicate, and *P* < 0.05 was considered to be statistically different. The graphs were plotted using GraphPad Prism 5.0 (GraphPad Software Inc., La Jolla, CA, USA).

## SUPPLEMENTARY MATERIALS



## Abbreviations

CRC: colorectal cancer
RGM: repulsive guidance molecule
BMP: bone morphogenetic protein
JNK: c-Jun-terminal kinase
p38 MAPK: p38 mitogen-activated kinase
Erk: extracellular signal-regulated kinase
PARP: Poly (ADP-ribose) polymerases
TUNEL: terminal deoxyribonucleotidyl transferase (TDT)-mediated dUTP-digoxigenin nick end labeling
Jam: junctional adhesion molecule
