# Supplementary material for: Dragon (RGMb) induces oxaliplatin resistance in colon cancer cells
Source: Oncotarget. 2016 Jun 30;7(30):48027–37. doi: 10.18632/oncotarget.10338 (PMC5216997; doi:10.18632/oncotarget.10338)
Supplement: Supplementary file 1 [file oncotarget-07-48027-s001.pdf]

Dragon (RGMb) induces oxaliplatin resistance in colon cancer cells

Supplementary Materials

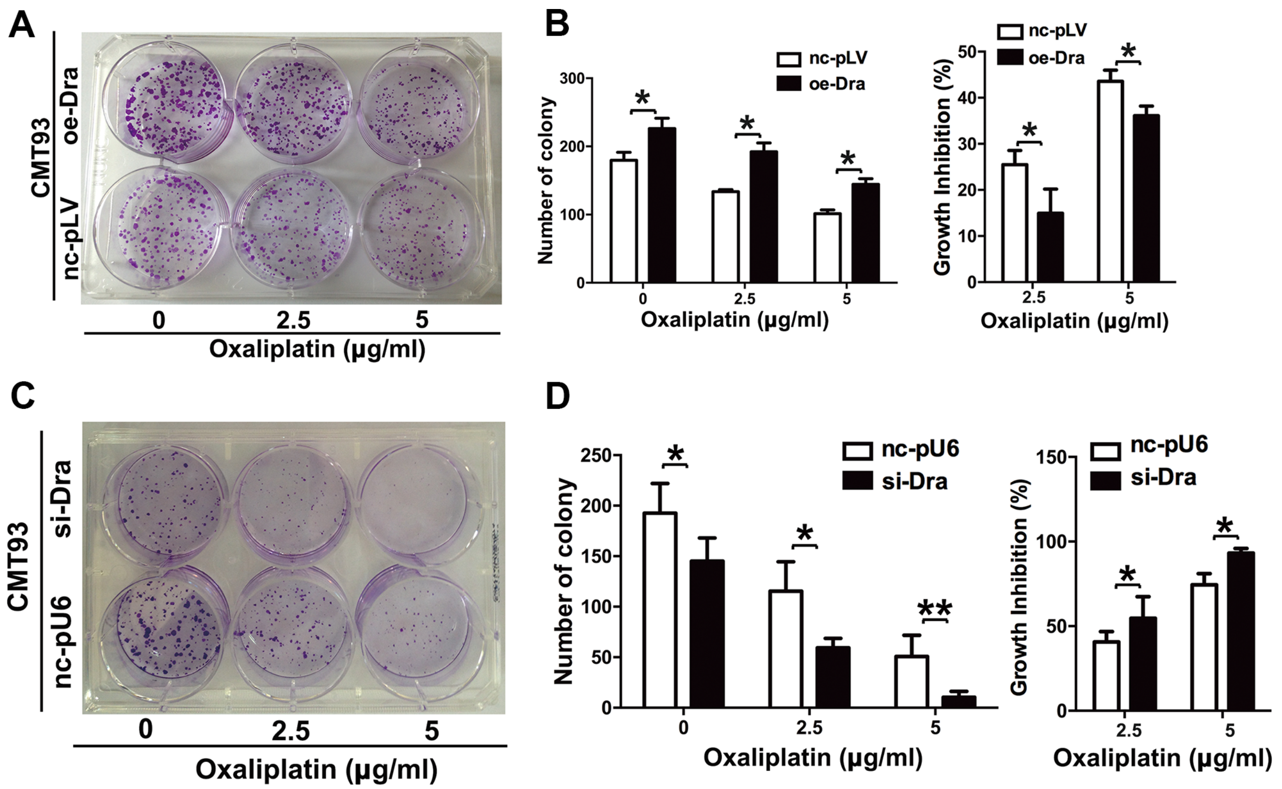

Supplementary Figure S1: Effect of Dragon on colony formation in the presence of oxaliplatin. (**A** and **B**) Control and Dragon-overexpressing CMT93 cells were used to perform the colony formation assay in the presence of increasing doses of oxaliplatin and high glucose for 7 days (A). The colony numbers (B, left panel) and growth inhibition rate (B, right panel) are presented. Only the colonies with more than 50 cells were counted. (**C** and **D**) Control and Dragon-knockdown CMT93 cells were used to perform the colony formation assay in the presence of increasing doses of oxaliplatin and high glucose for 7 days (C). The colony numbers (D, left panel) and growth inhibition rate (D, right panel) are presented. Only the colonies with more than 50 cells were counted. *$P < 0.05$ and **$P < 0.01$.

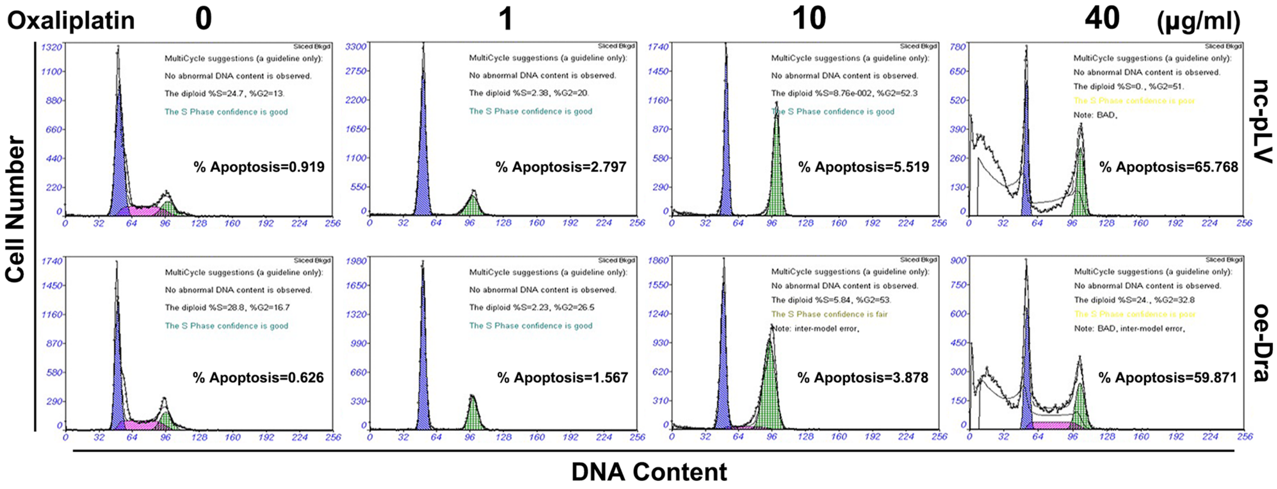

Supplementary Figure S2: Dragon overexpression inhibits oxaliplatin-induced apoptosis in HCT116 cells. Glucose-deprived control and Dragon-overexpressing HCT116 cells were treated with increasing doses of oxaliplatin for 24 h before the cells were stained with PI. Cells apoptosis was analyzed by flow cytometry.

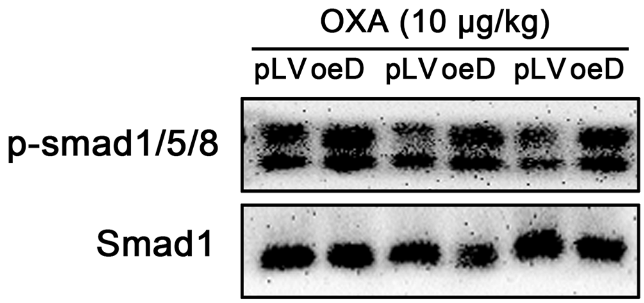

Supplementary Figure S3: Effect of Dragon overexpression on Smad1/5/8 phosphorylation in mouse xenograft tumors treated with oxaliplatin. Control (pLV) and Dragon-overexpressing (oeDra) xenograft tumors derived from oxaliplatin-treated CMT93 cells were collected for Western blotting to detect p-Smad1/5/8 and Smad1.

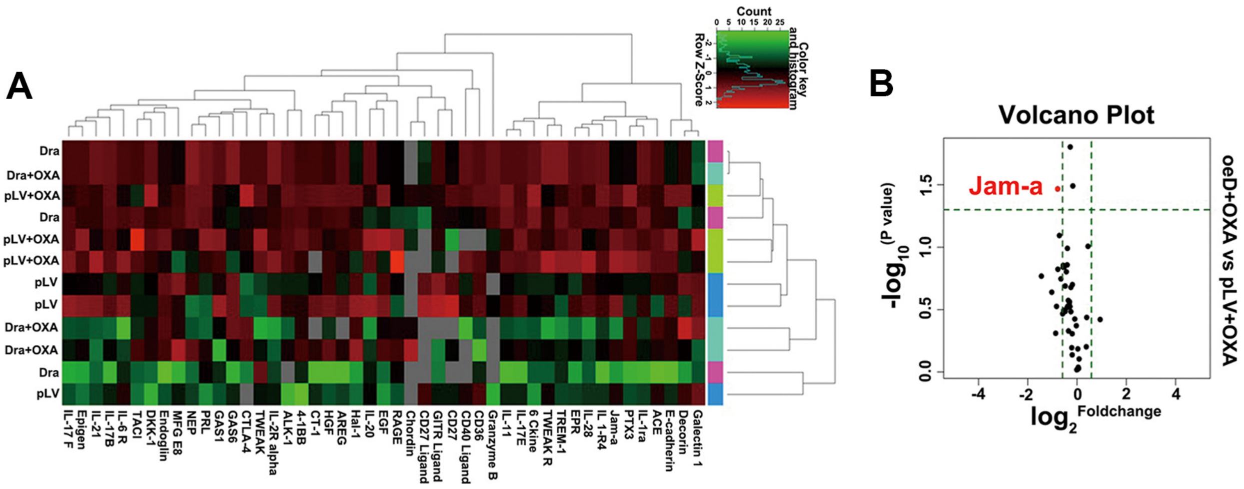

Supplementary Figure S4: Jam-a is downregulated in Dragon-overexpressing xenograft tumors. A cytokine antibody array was used to analyze the expression of 48 cytokines in oxaliplatin-treated xenograft tumors derived from control and Dragon-overexpressing cells. The heatmap of Hierarchical clustering analysis (**A**) and the volcano plot (**B**) showed that Jam-a was downregulated ($P < 0.05$) in Dragon-overexpressing xenograft tumors compared with controls treated with oxaliplatin.
